# Treatment of Aneurism by Iodide of Potassium

**Published:** 1869-12

**Authors:** 


### TREATMENT OF ANEURISM BY IODIDE OF POTASSIUM.

In Vol. 1, p. 253, of the *Gazette*, we reported 12 cases successfully treated. In the *Edinburgh Medical Journal*, for July, 1869, Dr. Balfour, of the Edinburgh Royal Infirmary, adds 11 additional cases, and says, that in all of them, there has been such a measure of success, as justifies him in saying this treatment holds out an excellent prospect of relief, and even of cure. He knows that spontaneous abatement occasionally takes place without any real improvement: but in all his cases, not only relief, but positive improvement was obtained in every instance. And he thinks that there are many facts which tend to prove that iodide of potash is not only curative in aneurism already developed, but that it also acts remedially and prophylactically in the aneurismal diathesis.

ANEURISM OF THE AORTA.

CASE I. Had been under observation for twelve months; the aneurism was reduced under treatment to a dull thud, in the second intercostal space, but no pain or uneasiness was complained of. The patient was able to tend shop.

CASE II. Aneurism of the abdominal aorta had remained quite well for more than a year after treatment.

CASE III. Aneurism of the innominata, implicating the carotid and subclavian arteries. There was also an aneurism of the abdominal aorta, and a general aneurismal condition of the arteries. He remained comfortable for many months after treatment. His abdominal aneurism could be felt as a hard, firm knot, much diminished in size; his innominate aneurism now never troubles him, but it is not absolutely consolidated, neither is it any longer a true aneurism; for it is restored to the condition of an elastic artery, enlarged fusiformly, of course, but no longer bulging as it formerly did, as a pulsating globular tumor stretching across the trachea.

CASE IV. Was an aneurism of the aorta, in a man aged 46. There was a large pulsating tumor to the right of the sternum, extending from the third to the seventh rib, and projecting fully one inch and a-half above the level of the ribs. Part of the tumor was solid; but all below the centre was soft and pulsated fluidly. The dullness extended fully five inches all around the centre of the tumor. He had intense dyspnœa, amounting to orthopnœa, violent, harassing, dry cough.

His food and drink were restricted in quantity; a belladonna plaster was put over tumor; 15 minims of chlorodyne were given every half hour to quiet his cough, and one tablespoonful of a solution of one-half ounce of iodide of potash in six ounces of infusion of chiratæ, was given in tablespoonful doses, three times a day. The patient was relieved in 48 hours and much improved in two months; the cough became very troublesome from time to time, but was relieved by the following prescription:—

℞. Morphiæ Hydrochlor, .................... gr. j.

Acid Hydrochlor, dilut., .................. m. v.

Acid Hydrocyan, ........................ ʒss.

Syrupi Scillæ, ........................... ℥j.

Aquæ Font, ............................. ℥j.

Given in teaspoonful doses every two or three hours.

Paralysis and loss of pulse in the left arm occurred and passed away. A violent serous diarrhœa was relieved by acetate of lead and opium, in small doses, without stopping the use of the iodide.

After five months treatment, the improvement was quite remarkable. The pulsation was very much lessened; the tumor was perfectly solid in every part and visibly decreased in size. The patient was still kept in bed, and the iodide of potash treatment still continued, in the hope of seeing the complete disappearance of this large tumor. But the patient obtained liquor on the sly, and relapsed; still, at the end of nine months, the tumor was considerably reduced in size, the cough was all gone, and the patient, although emaciated to a skeleton, was able to walk out daily.

Case V. Was one of aneurismal dilatation of the aorta. The patient (aged) had suffered for 16 months with severe pain across upper part of sternum, and breathlessness. There was dulness on percussion across the whole of the upper part of the breast-bone. There was a pulsating tumor deep in the tracheal fossa, and a double bruit, extending up into the left carotid. Six drachms of iodide of potash were dissolved in six ounces of infusion of chiratæ, and a tablespoonful given three times a day. The patient was kept in bed, and his food and drink restricted. He was quickly relieved; and in a fortnight the rasping bruit was softened, and the pulsation much lessened.

Case VI. Was one of aneurism of the aorta, which, taken singly, would attract much attention, but is remarkable as one of a series.

The patient, aged 40, had suffered for twelve months with a severe and constant aching pain in the chest. His breath was short and wheezing; cough troublesome, choking feeling on stooping, difficulty in swallowing solid food, pulse at left wrist almost imperceptible. There was a distinct bulging on the upper part of the left side of the chest, most marked over the second rib, and second intercostal space, also decided impulse, and dulness from the clavicle to within two inches of the nipple, over which a double blowing murmur was to be heard. There was also enlargement of the heart and valvular disease.

Pain and sense of choking prevented the patient from sleeping. Two drachms of iodide of potash were dissolved in six ounces of water, and two tablespoonfuls given three times a day, with chlorodyne, ether, and morphine injections, etc., to relieve the cough. In the course of six months, after much suffering from pain and cough, he was materially better, the breathing was easier, cough and expectoration almost gone; but slight fatigue or exposure to cold would bring them back again, and, at one time, there was complete loss of pulse in the left arm, with coldness and excruciating pain, the expectoration became copious, purulent, and bloody. He recovered from this, and during the whole of the next nine months continued to take two drachms of the iodide daily, with the exception of twice, when it caused pain, vomiting, and gastric irritation.

It was not till the end of 18 months that the tumor seemed sufficiently consolidated to allow the patient to get out of bed; and then, although he looked well and healthy, one drachm of iodide of iron was added to his medicine. There was great dulness on percussion of the chest, the sternal ends of both clavicles were dislocated, there was considerable puffiness and enlargement of the veins of the chest, a solid mass could be felt in the tracheal fossa, behind the sternum, over the left subclavian there was a solid tumor, pulsating not very forcibly, and only with a movement of elevation, but more of dilatation, over which no bruit could be heard, but only a dull thud. The patient had no difficulty of breathing or swallowing, could go up and down stairs and walk about, no cough, and aneurism evidently consolidated or consolidating. Dr. Bennett was also satisfied with the reality of the improvement.

Case VII. Aneurism of the aorta in a man aged 22, had lasted nine months, attended with harassing cough and copious purulent expectoration, severe pains in left side of chest and neck, and in the left arm. Pulse 110; heart normal; between second and third left ribs, there was a conical elevation, one inch and a-half long, rising half an inch above the level of the ribs, pulsating fluidly, with thin walls and distensile action. Dulness on percussion, extending from the first rib down toward the sternum, and over this space a pulsatile wave passed from right to left, attended with a tolerably loud and well-marked bruit.

The belladonna plaster, morphine mixture, and iodide of potash, one drachm to the ounce of water, was used in one-half ounce doses, *i.e.*, 30 grains per dose, three times a day. The pain ceased in a few days, in a month the cough was all gone, his breathing easy, and he comfortable. In a month more, the cough and expectoration had entirely ceased, and the patient thought himself cured. But the pulsating tumor, although lessened in size, had not thickened in its walls, and the bruit was as loud as ever. He left the hospital and relapsed; in a month, cough became harassing and he expectorated 15 ounces of purulent matter in one night. He required ten minims of chlorodyne to keep him from vomiting the iodide; but in six weeks more his cough and expectoration was all gone, he was looking well and gaining flesh, and the pulsations were quieter. At the end of four months the pulsations were so quiet, and the walls felt so solid and dense, that he was able to leave the hospital.

Case VIII. Was rather a case of disease of the aortic valves, attended with violent palpitation, severe pain over the heart, difficulty of breathing, with feeling of suffocation, on walking or going up stairs, all of which was relieved in a month by one-half drachm doses of the iodide, three times a day.

Case IX. Was an aneurism of the innominata, in a patient aged 37, sick for three months with beating in the throat, pains shooting down from there into the right shoulder and arm, and especially up the right side of neck and head. On the right of the tracheal fossa there was a tolerably firm, but distinctly expanding, pulsatile tumor, rising up out of the chest, and nearly two inches in diameter. There was dulness on percussion, a dull thud, but no bruit on auscultation, propagated up the carotids, but not along the subclavians. Pulse 120. He took one-half drachm of the iodide three times a day, for three months, when the tumor was quite firm and solid, no longer dilating, but not materially lessened in size; and nine months after he was able to work, the tumor having almost disappeared, although there was still excessive pulsation.

Case X. Was an aneurism of the aorta in a man aged 47, with cough, pain in the chest and down left arm, followed by great swelling of it; large pulsating tumor of right side. The patient had pain in the head and coryza, from the use of the iodide, but tolerance was soon established. Case is still under treatment.

Case XI. Was one of the aorta, which had been kept in abeyance for six years, with the iodide, taken of his own accord, while following his business as a peddler. He was then treated in hospital, by recumbent posture, restriction of food and drink, and drachm doses of the iodide, three times a day. In two months, he was much relieved, pulsations were no longer perceptible. Finally, while sinking from dropsy, he suddenly died of hemorrhage from the mouth. The heart and kidneys were sound, the aneurism did not press upon the trachea, bronchi, or gullet, the whole ascending aorta was atheromatous and calcareous; the middle coat greatly thinned; the mouth of the aneurism was three inches vertically and two inches across; the aneurism itself was four and a-half inches in breadth, and five inches long. A second aneurism of the size of a walnut rose from the right side of the aorta and pressed on the right auricle so as to occasion the dropsy. The larger aneurism contained firmly adherent fawn-colored clots, and some large, softer, and dark clots. The aneurism had not ruptured, and Balfour thinks it affords an apt illustration, not only of the mode of cure of an aneurism, but of one of those uncommon occurrences (the pressure of the right auricle), which rendered a well-devised and successful mode of cure abortive.—J. C. P.—*N. Y. Med. Gazette.*

